# Ethnicity-specific patterns of epigenetic age acceleration in rheumatoid arthritis

**DOI:** 10.1007/s11357-025-01508-w

**Published:** 2025-01-11

**Authors:** Archana Sharma-Oates, Niall Dunne, Karim Raza, Leonid Padyukov, Natalie Rivera, Annette van der Helm-van Mil, Arthur G. Pratt, Niharika A. Duggal, Simon W. Jones, Janet M. Lord

**Affiliations:** 1 School of Biosciences, University of Birmingham, Birmingham, UK; 2 Institute of Inflammation and Ageing, University of Birmingham, Birmingham, UK; 3 MRC-Versus Arthritis Centre for Musculoskeletal Ageing Research, University of Birmingham, Birmingham, UK; 4 NIHR Birmingham Biomedical Research Centre, University Hospital Birmingham and University of Birmingham, Birmingham, UK; 5 Department of Rheumatology, Sandwell and West Birmingham NHS Trust, Birmingham, UK; 6 Karolinska Institute, Stockholm, Sweden; 7 University of Leiden, Leiden, the Netherlands; 8 Translational and Clinical Research Institute, Newcastle University, Newcastle Upon Tyne, UK; 9 Department of Rheumatology, Newcastle Upon Tyne Hospitals NHS Foundation Trust, Newcastle Upon Tyne, UK

**Keywords:** Rheumatoid arthritis, Ethnicity, DNA methylation, Epigenetic clock, South Asian

## Abstract

Rheumatoid arthritis (RA) is an age-related chronic inflammatory disease which may include accelerated biological ageing processes in its pathogenesis. To determine if increased biological age is associated with risk of RA and/or is present once disease is established. We used DNA methylation to compare biological age (epigenetic age) of immune cells in adults at risk of RA and those with confirmed RA, including twins discordant for RA. The established RA studies were secondary analyses of existing DNA methylation data. Sub-group analysis considered the influence of ethnicity. Four epigenetic clocks were used to determine DNA methylation age. DNA methylation age was no different in adults at risk of RA in the Leiden Clinically Suspect Arthralgia (CSA) cohort (*n* = 38 developed RA, *n* = 24 did not), and there was also no difference in DNA methylation age between 77 UK twins discordant for RA, or adults with established RA from the Swedish EIRA cohort (*n* = 342) compared to healthy controls (*n* = 328). A sub-group analysis of RA patients of South Asian ethnicity (10 RA patients, 14 healthy controls) showed DNA methylation age acceleration of 3.3 years (*p* = 0.00014) using the mean DNA methylation age of four epigenetic clocks. Our study suggests that epigenetic age acceleration may be differentially influenced by South Asian ethnicity, but that RA was not generally associated with accelerated epigenetic age. The higher epigenetic age in the South Asian patients may explain the earlier age of onset in this minority ethnic population.

**Supplementary Information:**

The online version contains supplementary material available at 10.1007/s11357-025-01508-w.

## Introduction

Advancing age is a major risk factor for several immune mediated inflammatory diseases including rheumatoid arthritis (RA) [1]. Despite the association of RA with age, we understand little of the role ageing processes play in disease pathogenesis. In the last decade, a degree of consensus has arisen around the core mechanisms that drive the aged phenotype, which have been termed the hallmarks of ageing [2]. The 12 hallmarks are proposed to act in series initiated by damage to DNA or proteins or epigenetic modifications that then result in cell responses such as compromised mitochondrial function, altered nutrient sensing and cell senescence. The final effector phase includes increased inflammation, in part driven by the pro-inflammatory phenotype of senescent cells, which has pleiotropic and negative impacts on body tissues [3].

It has been shown that ageing mechanisms such as cell senescence are heightened in a range of age-related diseases such as osteoarthritis [4], chronic kidney disease [5] and frailty [6]. The possibility exists that RA pathogenesis may include a role for the biological ageing process. For example, it has been known for over two decades that ageing of the immune system, termed immunesenescence, is seen in patients with established RA [7]. Crucially, key features of immunesenescence, such as thymic atrophy [8], lymphocyte telomere shortening [9] and dysregulated Treg and Th17 responses [10], occur much earlier in RA patients than age-matched healthy adults. Other hallmarks of ageing have been detected in synovial fibroblasts from RA patients including damaged mitochondria [11] and cell senescence [12].

Several questions arise from the current literature: Is the presence of several of the hallmarks of ageing in RA, both in immune cells and stromal cells in affected joints, indicative of a broader acceleration of biological age? If this is true then is this a risk factor in RA, akin to smoking status, occurring prior to disease onset, or is it a consequence of RA and particularly the chronic inflammatory state seen in the disease? If it is a primary pathogenic factor, targeting ageing processes, for example through the use of senolytic agents [13], could reduce the risk of progression from at risk stages to the development of established RA. If biological age acceleration is a consequence of disease, this is still important information as targeting the process could also deliver benefits in those with established disease, for example by suppressing the proinflammatory actions of senescent immune cells [14] with senomorphic drugs, or by reducing epigenetic age to reduce risk of other age-related diseases [15].

These questions can now be addressed as there are several biomarkers that appear to indicate an individual’s biological as opposed to their chronological age. One of the most widely used are the epigenetic clocks based upon DNA methylation analysis, where the degree of methylation at specific sites in the genome correlates with chronological age [16]. Deviation from this association, for example higher methylation than predicted, is associated with increased mortality risk and therefore potentially a higher biological age. The original clocks were developed by Horvath [17] and Hannum [18], the former is not tissue specific whereas the latter is based upon blood cell DNA methylation data.

To determine if and at what disease stage biological age acceleration occurs in RA, we calculated DNA methylation age in cohorts at risk of RA, including twin pairs with an affected and unaffected sibling, and in patients at different points in the pathway to established RA. Specifically, we have assessed DNA methylation in blood cells from adults showing clinically suspect symptoms (arthralgia) but without arthritis and those with RA confirmed by the 2010 ACR/EULAR criteria [19] but who had not yet commenced on DMARD therapy. We have focussed upon ACPA positive RA to reduce heterogeneity and increase the chance that we were studying disease with similar pathogenic mechanisms.

## Methods

### Participants

Different cohorts of patients were used to determine at which stage any DNA methylation differences might occur. A cohort from Leiden provided samples, this cohort was one at risk of developing RA, either from demographic, clinical (presence of arthralgia) or genetic data. The Birmingham Early Arthritis Cohort (BEACON) [20] and the EIRA cohort of Swedish patients [21] with diagnosed RA by ACR/EULAR 2010 criteria provided samples and data for DMARD naïve patients. All participants gave written informed consent and all three studies had ethics approval: the Leiden cohort study was approved by the LUMC Medical Ethical committee, the BEACON study was approved by the Black Country Research Ethics committee (Ref: 12/WM/0258) and the EIRA study was approved by the Karolinska Institute ethical review board. We also accessed existing DNA methylation data from the TwinsUK [22] and Manchester Twins [23] cohorts.

### DNA methylation analysis

Stored samples of peripheral blood mononuclear cells (PBMCs) were used for DNA methylation analysis. DNA was extracted using a DNeasy Blood and Tissue kit (Qiagen, Hilden, Germany) and DNA integrity was determined using BioAnalyser. Bisulphite conversion was performed using Zymo EZ DNA methylation kits following manufacturer’s instructions (Zymo Research, Freiburg, Germany). DNA methylation was measured by the Birmingham Genomics facility using the Illumina Infinium Methylation EPIC BeadChip (Illumina, San Diego, CA).

### DNAm data pre-processing

Data pre-processing steps included removal of probes overlapping single nucleotide polymorphism (SNPs) with minor allele frequency > 1%, according to the NCBI Database (dbSNP). The SNP annotation was obtained from the ‘IlluminaHumanMethylation450kanno.ilmn12.hg19’ Bioconductor R package for Illumina’s 450 K methylation arrays. The data was Noob normalised and all pre-processing was done using the R minfi package, version 1.38.0.

Methylation at specific sites was calculated as *β* = max(M,0)/[max(M,0) + max(U,0)], where max(M,0) is the fluorescence intensity of methylated (M) alleles (signal A) and max(U,0) is the fluorescence of un‐methylated (U) alleles (signal B). Thus, *β* values range from 0 (completely un‐methylated) to 1 (completely methylated).

Epigenetic age (DNA methylation age) was calculated from the DNA methylation data using published algorithms, namely those produced by Horvath [17, 24] and Hannum [18]. DNA methylation age was predicted based on regression coefficients from several training sets. Additionally, the PhenoAge [25] and GrimAge epigenetic clocks were also calculated as indicator of mortality and healthspan [26].

Secondary analysis of two sets of existing DNA methylation data were also analysed using the same algorithms from the following: 1. Swedish patients with established RA and age-matched healthy controls in the EIRA cohort (accession GSE42861) using demographic data provided the authors; 2. A UK cohort of RA discordant monozygotic twins (accession E-MTAB-6988).

### Statistical analysis

All analyses were performed in the R statistical programming environment, R version 4.1.0. Epigenetic age was calculated using the R dnaMethyAge package, version 0.2.0 [27]. Age acceleration was determined by the residual of the difference between the estimated epigenetic age and the chronological age. Positive residual values indicated an individual was biologically older than their chronological age and the reverse for negative residual values. Multiple regression analysis was used to explore the relationship between predicted epigenetic age and covariates such as gender and disease status. Robust regression models were generated for datasets that were not normally distributed. The *p* values for statistical comparisons between the mean of chronological age and the predicted epigenetic age were generated using the method wilcox.test of stats_compare function in R.

## Results

### DNA methylation age in patients at risk of developing RA (Leiden CSA cohort)

**Fig. 1:**
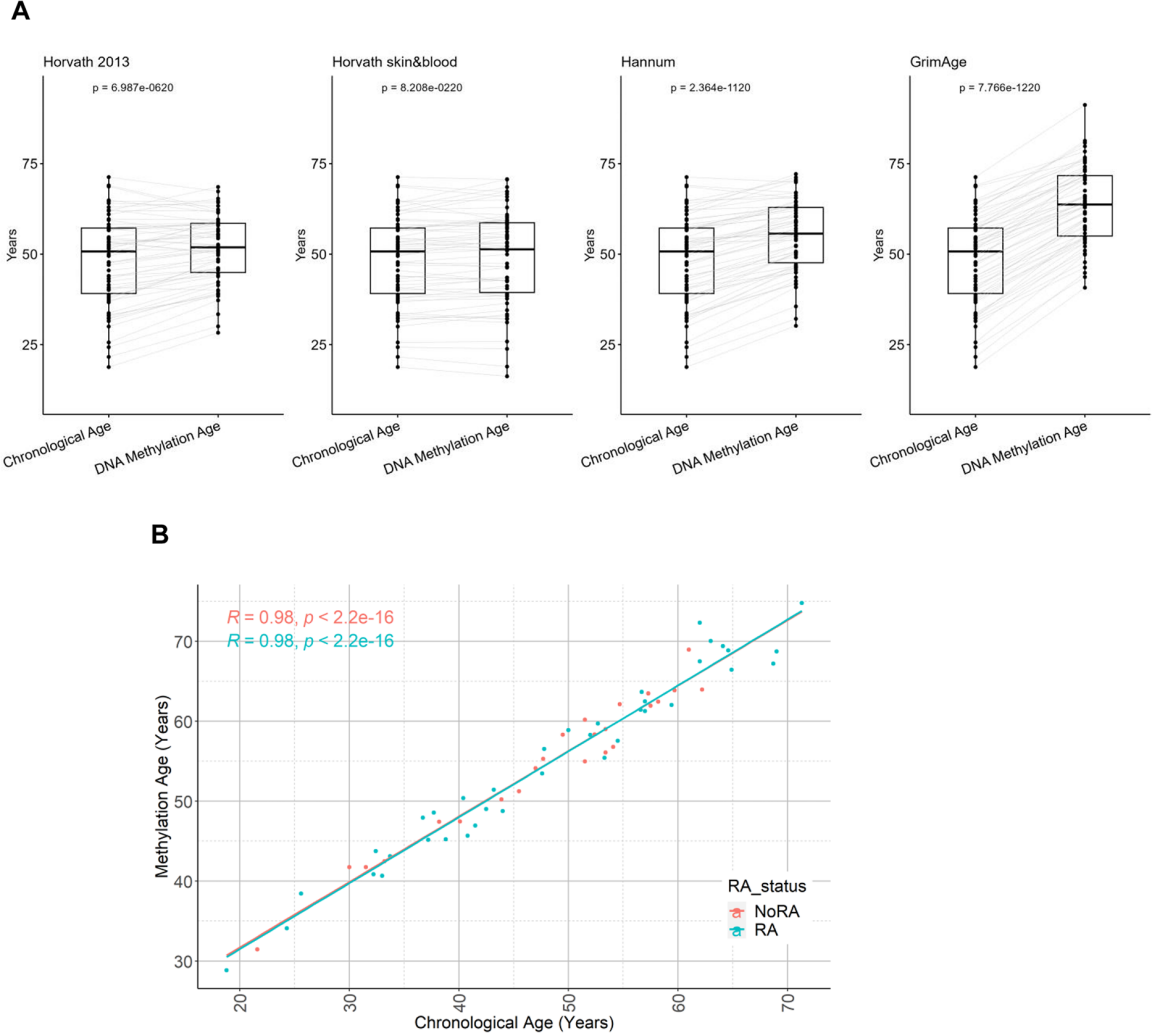
DNA methylation age in a cohort at risk of RA. **A** Sankey box plots demonstrating an increased methylation age in the Leiden cohort using the Horvath multi-issue, Hannum and GrimAge clocks, but no difference detected using the Horvath skin and blood clock. **B** No difference was observed between the predicted epigenetic ages of patients with RA and those who did not develop RA within the Leiden cohort. The regression plot was generated using the mean value of epigenetic age determined using the Hannum, Horvath multi-tissue, Horvath skin and blood and GrimAge clocks

We first carried out DNA methylation analysis of PBMC-derived DNA samples from patients at risk of developing RA. The demographic data of the cohort with Clinically Suspect Arthralgia (CSA) are shown in Table 1. Using the Leiden CSA cohort of 62 at risk individuals, 38 who subsequently developed RA and 24 who did not, we showed a good correlation between chronological age and DNA methylation age (Supplementary Fig. 1a). We found increased DNA methylation age compared with chronological age by three of the epigenetic clocks, namely the first-generation Horvath and Hannum clocks and the GrimAge clock, but not with the second-generation Horvath Skin&Blood clock (Fig. 1A). However, there was no difference in the increase in epigenetic age, using the averaged data of these four clocks, between the at-risk group who developed RA and those who did not (Fig. 1B).

**Table 1:** Study characteristics for each of the cohorts

	Study Cohorts		
Description	Leiden CSA	EIRA	Discordant twins
Total participants (*n*)	62	670	144
Age (years) median (IQR)	50.8 (39.1–57.2)	54.0 (45.0–61.0)	56.0 (47.0–64.0)
Female *n* (%)	50 (80.6)	479 (71.5)	132 (91.7)
Male *n* (%)	12 (19.4)	191 (28.5)	22 (15.3)
Disease status			
At risk of RA	38	NA	NA
Established RA, DMARD naive	38	342	72
Healthy control	NA	328	72

### DNA methylation age in discordant twins and non-twin RA patients

**Fig. 2:**
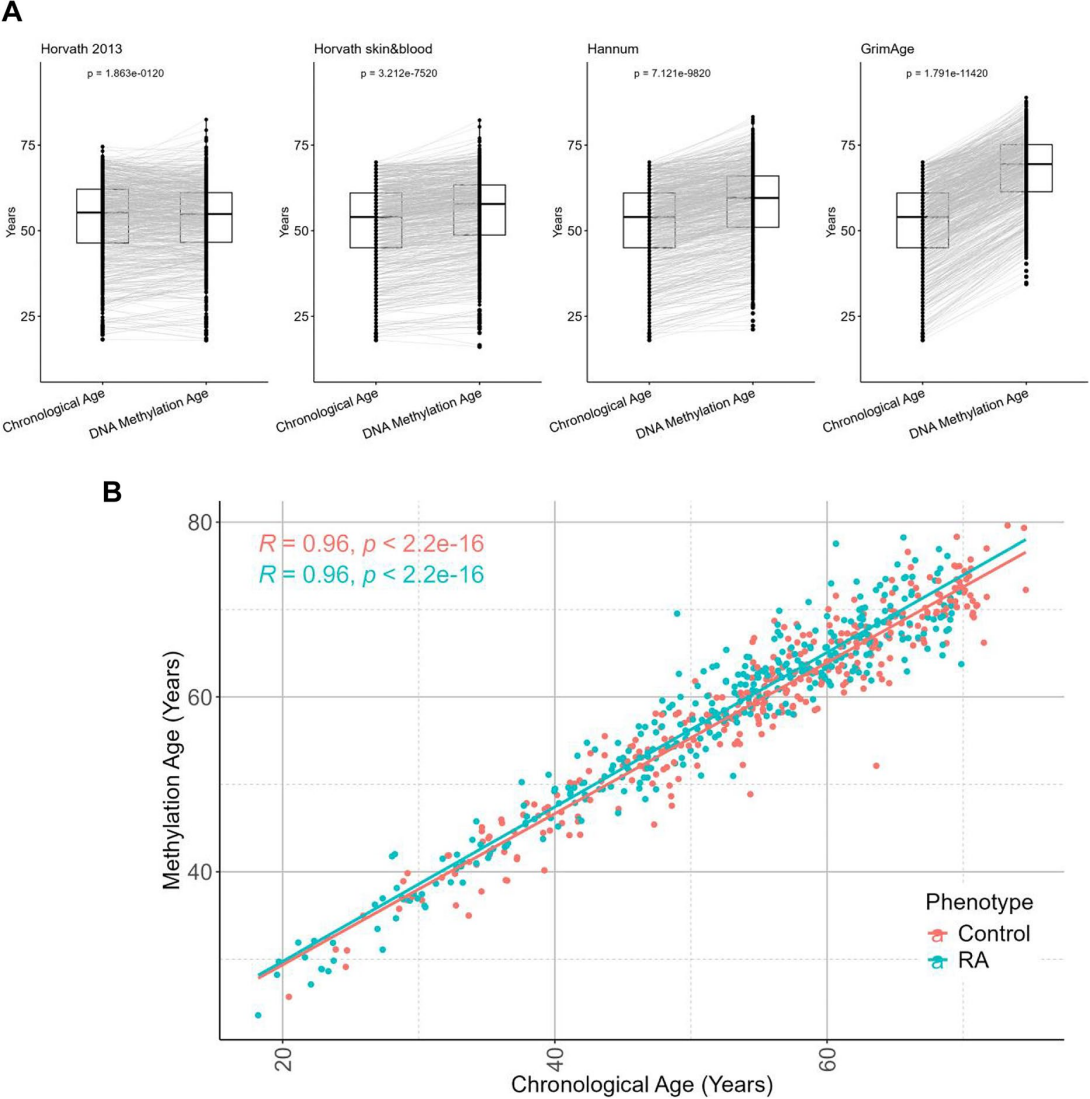
DNA methylation age in a cohort of adults with confirmed RA.** A** Sankey box plots showing significantly higher methylation age detected in both DMARD naïve RA patients and controls within the EIRA cohort using the four epigenetic clocks, Horvath multi-tissue, Horvath skin and blood, Hannum and GrimAge, respectively, from left to right. **B** The regression plot shows no difference observed between the predicted epigenetic ages of participants with RA and controls from the EIRA cohort. The plot was generated using the mean value of epigenetic age determined using the Hannum, Horvath multi-tissue, Horvath skin and blood and GrimAge clocks

To assess whether DNA methylation age might be increased in patients with established RA and may thus be a consequence rather than a cause of disease, we carried out DNA methylation analysis of patients recently diagnosed with RA but who had not yet begun on DMARD therapy, using existing data from the Swedish EIRA cohort. Analysis of data from this cohort of 342 RA patients and 328 healthy controls, demographic data shown in Table 1, revealed DNA methylation ages that were higher than chronological age by the four different clocks used (Fig. 2A). Again, the epigenetic age acceleration was seen in both the RA and control groups, when the average of the four clocks was used (Fig. 2B).

**Fig. 3:**
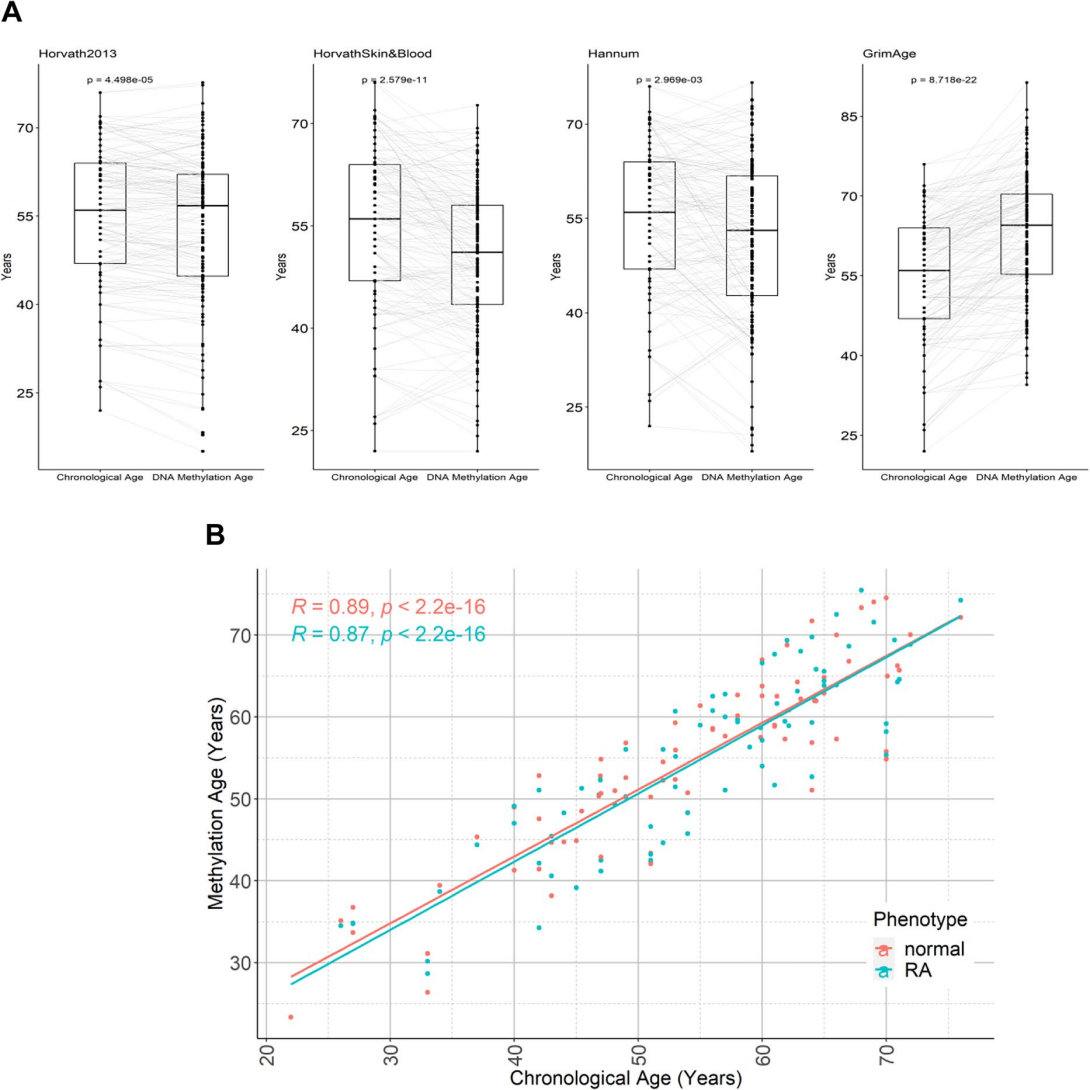
DNA methylation age in a cohort of UK twins discordant for RA. **A** Sankey box plots showing comparisons between chronological age and predicted methylation age using Horvath multi-tissue clock, Horvath skin and blood, Hannum and GrimAge for all participants in the discordant twin cohort. **B** The regression plot shows no difference observed between the predicted epigenetic ages of discordant twins with or without RA. The plot was generated using the mean value of epigenetic age determined using the Horvath multi-tissue clock, Horvath skin and blood, Hannum and GrimAge clocks

We next carried out secondary analysis of another existing epigenetic data set for 77 UK twins discordant for RA; the demographic data for the cohort are shown in Table 1. The data showed good correlation between DNA methylation age and chronological age (Supplementary Fig. 1b), and there were differences between chronological age and DNA methylation age across four epigenetic clocks (Fig. 3A). Again, there was no significant difference in DNA methylation age acceleration between the RA and non-RA twins using the mean of the four different epigenetic clock algorithms used (Fig. 3B).

### DNA methylation in South Asian RA patients

One unexpected finding arose from the ethnic diversity seen in a fourth cohort of patients from the Birmingham BEACON cohort of DMARD naïve RA patients. Ten of this cohort were of South Asian (SA) ethnicity and we compared their DNA methylation age with 14 healthy South Asian adults recruited from the community. Approximately 83% of the cohort were female (Table 2) and the mean age was 41.0 years (SD = 9.98). Of the 10 South Asian RA patients, nine had never smoked, none of the healthy group were smokers. Six (60%) of the RA group were obese or overweight, but none of the healthy group were in the obese or overweight category (Table 2).

**Table 2:** Study characteristics of South Asian cohort

Description	Healthy	Established RA, DMARD naive	Total
Participants (*n*)	14	10	24
Age (years) median (IQR)	40.0 (37.0–48.75)	48.0 (36.3–53.8)	41.0 (37.0–50.5)
Female *n* (%)	12 (86)	8 (80)	20 (83.3)
Male *n* (%)	2 (14)	2 (20)	4 (16.7)
Smoking *n* (%)	0 (0)	1 (0.1)	1 (0.04)
BMI			
Underweight (< 18.5) *n* (%)	0 (0)	1 (10)	1 (0.04)
Healthy (18.5–24.9) *n* (%)	7 (0.5)	3 (30)	10 (0.42)
Overweight (25–29.9) *n*(%)	6 (0.43)	3 (30)	9 (0.38)
Obese (≥ 30), *n* (%)	1 (0.07)	3 (30)	4 (0.17)

**Fig. 4:**
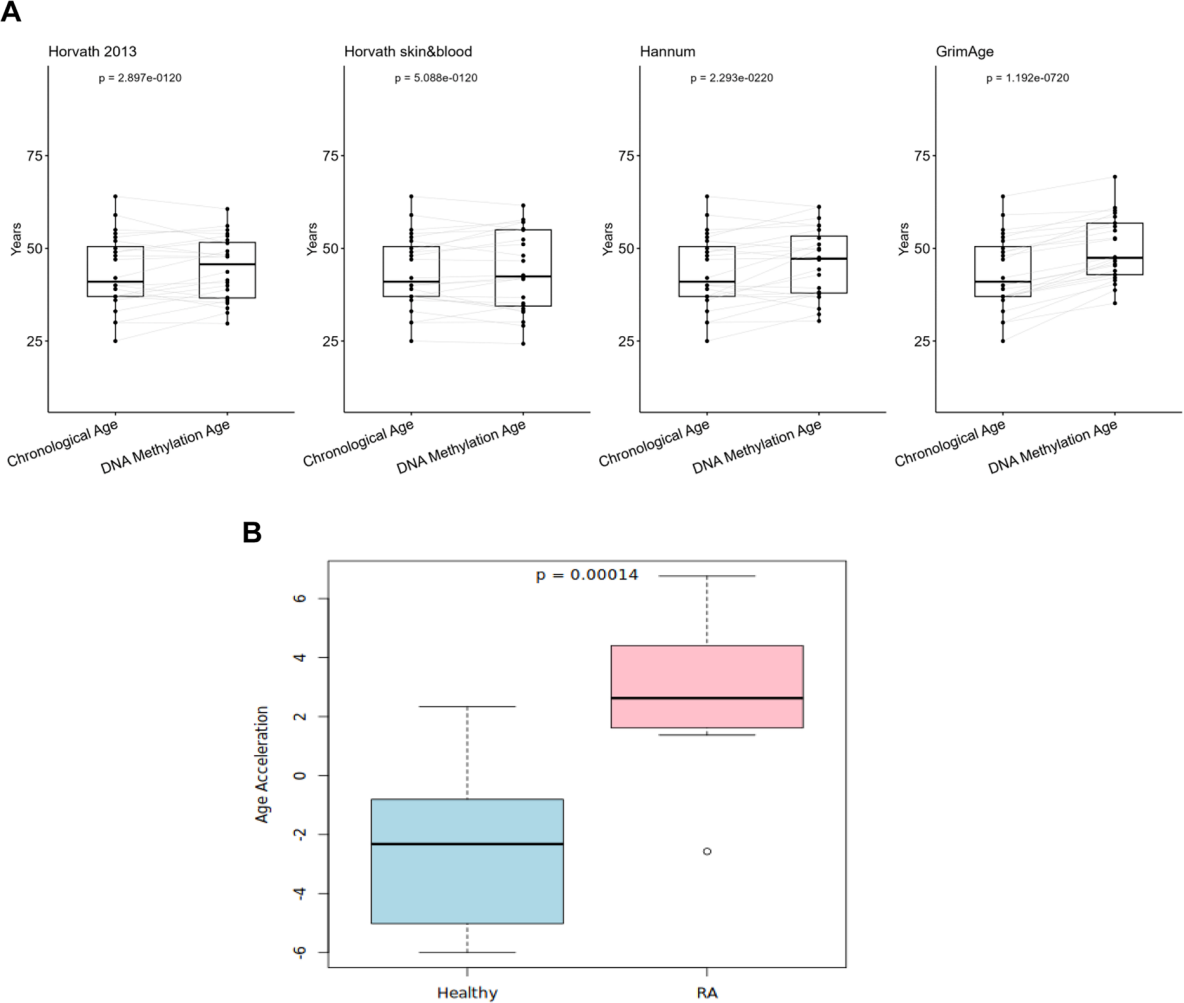
DNA methylation age in a cohort at UK resident South Asian RA patients. **A** Sankey plot showing the predicted methylation age as compared to chronological age in 10 South Asian patients with RA, using each of the four clocks individually, Horvath multi-tissue, Horvath skin and blood, Hannum, and GrimAge clocks, respectively, from left to right. A significantly higher methylation age was only detected with the Hannum and GrimAge clocks. **B** The box plot shows a significant difference detected in the mean DNA methylation age predicted by the four clocks between the South Asian healthy controls and South Asian RA patients

Epigenetic age was higher than chronological age in the SA cohort overall by the Hannum and GrimAge clocks, but this did not reach significance for the two Horvath clocks (Fig. 4A). There was a significant difference in DNA methylation age acceleration between the RA and non-RA SA participants using the mean of the four different epigenetic clock algorithms used (Fig. 4B). These data were interesting as it suggested that accelerated ageing was a feature of RA in this ethnic group. A multiple regression analysis of the cohort, taking smoking and BMI status into account, showed the RA patients who had never smoked and adjusted for obesity, had an acceleration in their biological age (the difference between their chronological age and epigenetic age) of 3.3 years (*p* = 0.00014) using the mean methylation age of the four clocks.

## Discussion

Here, we investigated the possibility that the age-related disease rheumatoid arthritis (RA) may include acceleration of biological ageing processes in its pathogenesis, based upon observations of increased hallmarks of ageing, such as cell senescence, in RA patient tissues [12]. Rather than focus upon distinct ageing processes^2^ we assessed the overall degree of biological ageing using DNA methylation based epigenetic clocks in DNA from PBMCs [17, 18]. Assessing epigenetic age (DNA methylation age) in patients at risk of developing RA, as well as those with established RA, revealed no acceleration of DNA methylation age associated with RA. There is thus an apparent discrepancy between reports of increased ageing in different tissues, such as greater immunesenescence in patients with RA [7–10] and the presence of different hallmarks of ageing in synovial fibroblasts from RA joints [11, 12]. This may simply reflect the inability of these DNA methylation based biomarkers to capture all aspects of cellular ageing; indeed, studies have shown that the blood based epigenetic clocks are not influenced by cellular senescence or telomere attrition [28] which are features of an aged immune system and have been reported as present in RA patients [11, 12]. It is also possible that biological ageing is occurring but is targeted at specific organs, articulated joints in the case of RA, and does not impact on broader physiological ageing. To support this argument, in vitro studies have shown that inflammatory cytokines can induce cell senescence in a variety of cell types [29, 30], which may explain the presence of senescent synovial fibroblasts in RA joints [12] without increased overall epigenetic ageing in PBMCs. Additionally, chronic inflammation induces senescence of synovial fluid mesenchymal stem cells in RA patients, which reduces their immunomodulatory properties [31] contributing to maintained inflammation in the joint. This further supports the importance of localised inflammation and tissue specific generation of hallmarks of ageing in the pathogenesis in RA.

The lack of any accelerated DNA methylation age in blood cells in a chronic inflammatory immune–mediated disease such as RA is still surprising as raised inflammation, one of the hallmarks of ageing^2^, has been associated with increased epigenetic age, explaining up to 11% of the increase in DNA methylation age in a 13-year longitudinal study of 940 participants aged 40–69 years at baseline [32]. One explanation is that inflammation is now well controlled in RA patients on DMARDs, thus removing one of the systemic drivers of higher epigenetic age. However, as our study included patients who were DMARD naïve this seems to be an unlikely explanation.

There are clear ethnic differences in the prevalence of non-communicable diseases, with higher incidence of cardiovascular disease [33] but lower cancer risk in SA populations compared with those of European ancestry [34]. These differences are only partly explained by genetic and environmental factors [33, 35], with epigenetic influences likely to play a role. Indeed, Epigenome Wide Association Studies (EWAS) of two cohorts with significant ethnic minority participants revealed that DNA methylation differences between ethnicities were widespread throughout the genome (3.4% of sites tested). Interestingly, a majority of associations were attributable to ethnic differences in cell composition with fewer effects related to smoking or genetic variation [35]. Although our sub-group analysis of SA RA patients was small, *n* = 10, it revealed that the South Asian RA patients had higher epigenetic age acceleration than non-RA SA controls, raising the possibility that the SA RA population may be ageing at a faster rate than European patients. Ethnicity differences in DNA methylation age acceleration have been reported, with those of Hispanic ancestry having lower rates than Europeans and East Asians showing no difference to those of European ancestry [36]. A comparison of DNA methylation age between UK resident South Asians and Europeans also showed a higher epigenetic age for the SA group by four out of five epigenetic clocks generated [37]. If epigenetic age acceleration differs with ethnicity, then we would predict that immune mediated inflammatory diseases (IMIDs), including RA, may occur at an earlier age in South Asian and other ethnic minority populations. In support of this suggestion, we have recently analysed UK primary care data and UK Biobank data comparing the age of onset of a range of IMIDs across different ethnic groups (South Asian, African-Caribbean, Mixed other, White). This analysis revealed an age of IMID onset that was earlier in the different ethnic minority groups compared to the White patients by between 2 and 30 years. Fibromyalgia was used as a non-inflammatory disease comparator and showed minimal difference in age of onset [38]. Further research to determine if differential epigenetic age is influencing the development of RA and other IMIDS is clearly required.

## Conclusions

Our study revealed that epigenetic age was differentially influenced by South Asian ethnicity, but that RA was not associated with accelerated epigenetic age, at least in immune cells. The higher epigenetic age in the SA RA patients may explain not only the higher prevalence of the disease but also its earlier age of onset in this population. As there are now studies showing that epigenetic age can be reversed [39], this may offer targeted new approaches to the treatment of RA in the future if these observations can be confirmed and if they can be targeted either at specific populations, or at tissues showing signs of increased hallmarks of ageing, such as the RA joint.

## Supplementary Information

Below is the link to the electronic supplementary material.

Supplementary Figure 1. Correlation between DNA methylation age and chronological age. Correlation plots for DNA methylation using the Horvath multi-tissue (Horvath2013_mAge) and Hannum blood and the Horvath skin and blood clocks (Horvath2018_mAge) and chronological age for (a) the Leiden CSA cohort and (b) the discordant twins cohort. (PPTX 704 KB)

## Data Availability

The primary data in this study are available upon reasonable request to the corresponding author.
