# Supplementary figures and images for: Ethnicity-specific patterns of epigenetic age acceleration in rheumatoid arthritis
Source: GeroScience. 2025 Jan 11;47(3):3965–75. doi: 10.1007/s11357-025-01508-w (PMC12181512; doi:10.1007/s11357-025-01508-w)

## Slide 1
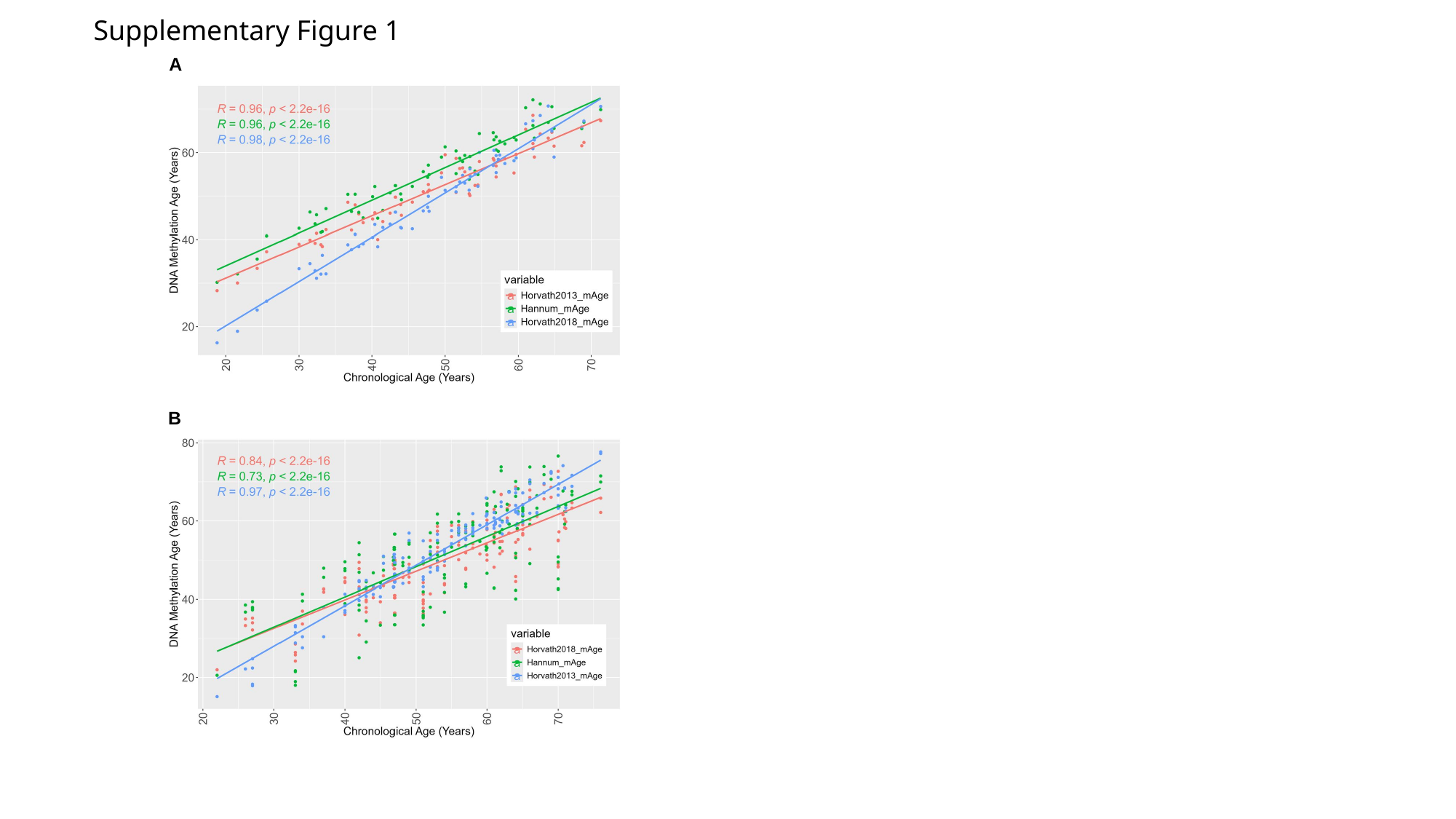

Supplementary Figure 1
A
B

Supplement: Supplementary file 1 — Supplementary Figure 1. Correlation between DNA methylation age and chronological age. Correlation plots for DNA methylation using the Horvath multi-tissue (Horvath2013_mAge) and Hannum blood and the Horvath skin and blood clocks (Horvath2018_mAge) and chronological age for (a) the Leiden CSA cohort and (b) the discordant twins cohort. (PPTX 704 KB) [file 11357_2025_1508_MOESM1_ESM.pptx]
